# Comparative study on lesions of reproductive disorders of cows and female dromedary camels slaughtered at Addis Ababa, Adama and Akaki abattoirs with bacterial isolation and characterization

**DOI:** 10.1186/s12917-021-02822-z

**Published:** 2021-03-29

**Authors:** Aynalem Mandefro Getahun, Gemechu Chala Hunderra, Tadesse Gidey Gebrezihar, Bulto Giro Boru, Natnael Teshager Desta, Tilaye Demissie Ayana

**Affiliations:** 1 Faculty of Veterinary Medicine, Hawassa University, Hawassa, Ethiopia; 2 College of Veterinary Medicine, Addis Ababa University, Addis Ababa, Ethiopia; 3 National Animal Health Diagnostic and Investigation Center, Sebeta, Ethiopia

**Keywords:** Cow, Dromedary camel, Lesion, Reproductive organ

## Abstract

**Background:**

Reproduction is a basic prerequisite to efficient livestock production. Reproductive performance depends on the normal structure and function of genital organs. A cross-sectional study was conducted from November 2016 to May 2017 to identify and compare the frequency of reproductive tract pathological lesions and to isolate bacteria associated to uterine lesions in female dromedary camels and cows slaughtered at Akaki camel slaughterhouse and Addis Ababa and Adama municipal abattoirs. Purposive sampling technique was employed to include and examine the reproductive tracts of all slaughtered animals (280; 140 cows and 140 camels) during the study period.

**Result:**

The study examined a total of 280 (140 cows and 140 camels) reproductive tracts. Various pathological lesions with different degrees of severity were observed in 48 (34.2%) and 51 (36.4%) of dromedary camels and cows, respectively. In dromedary camels, the most prevalent lesion was uterine lesions (21.4%) followed by ovarian lesions (7.14%); while in cows, ovarian lesions were the most prevalent (16.4%) followed by uterine lesions (14.2%). In general, 56 bacteria were isolated from cows’ uterine lesion, the *Staphylococcus* species (28.5%), *Streptococci* species (19.6%), *Coynebacterium* species (8.9%), *Escherichia coli* (26.78%), *Salmonella* species (10.7%) and *Klebsiella* species (5.35%) being the most representative isolates. In camels, however, 45 bacteria were isolated from uterine lesions with higher prevalence of *Escherichia coli* (35.5%), *Staphylococcus* species (26.6%), *Streptococcus* species (13.3%), *Pseudomonas* species (6.6%), *Proteus* species (4.4%), *Salmonella* species (8.8%) and *Klebsiella* species (4.4%). Bacteriological data showed that the major isolates were similar, although slightly more frequent in occurrence in cows. Microscopically, uterine inflammatory lesions evidenced endometrial glands degeneration, epithelium sloughing, peri-glandular cuffing, and inflammatory cells infiltration.

**Conclusions:**

In female dromedary camels and cows, pathological lesions of the reproductive tract showed great prevalence, with similarity in bacterial isolates in both species. The role of each reproductive lesion and bacterial isolates as causal agents of reproductive failures in these livestock species, however, needs further investigation.

**Supplementary Information:**

The online version contains supplementary material available at 10.1186/s12917-021-02822-z.

## Background

Reproduction is a basic prerequisite to efficient livestock production. Productivity of animals largely depends on their reproductive performance; hence regular and successful reproduction is a key to profitable animal production. Large animals that rarely deliver a live calf are not worth keeping [1]. Therefore, reproductive efficiency is an important asset for achieving maximum return from the animal [2]. Reproductive performance of a given animal depends upon the normal structure and functions of genital organs [3]. However, the physiological process of reproduction could get disrupted due to a variety of factors like nutritional deficiencies, various diseases, hormonal abnormalities, and environmental stress [4]. Thus, identification of reproductive tract abnormalities is important, especially, when dealing with genetically superior animals [5]. Reproductive diseases are important contributor to the decline in fertility potential of large farm animals. Generally, in large dairy farm animals, infertility might result in decreased milk production, treatment and labor costs, and increased rate of culling [6].

Pathological lesions of genital tracts are believed to be the major reason for economic losses associated with infertility, culling and slaughtering of cows [7–9] and female dromedary camels [10, 11]. In general, female livestock animals are culled because it is either uneconomic to maintain them or they have chronic or untreatable diseases. Hence, abattoirs are good sources, for studying pathological lesions of reproductive organs that are severe enough to cause infertility and even sterility [12]. Moreover, most female reproductive organ pathological lesions lack additional outward manifestations in which case most of these abnormalities can only be diagnosed when the animal is subjected to postmortem examination [13]. Thus, examination of gross and microscopic lesions of genital tract play a central role in the identification of these problems.

Previously, abattoir-based studies on reproductive organs abnormalities of cows have been documented elsewhere in Ethiopia [1, 7–9, 14]. According to these studies, ovariobursal adhesion, follicular cyst, luteal cyst, paraovarian cyst, ovarian hypoplasia, vaginitis, cervicitis, hydrosalpinx, pyosalpinx, hydrometra, endometritis, cervical ring hypoplasia and hypoplasia of the uterus were some of the pathological lesions recorded. Similar findings were also been reported from other parts of the world [15–17]. In Ethiopia, however, limited documented information is available regarding female dromedary camels’ reproductive organs lesions [11]. Nevertheless, studies from other parts of the world have reported different reproductive pathological lesions of uterus, ovary, oviduct, cervix and vagina of which lesions of uterus were the most frequent in almost all studies [6, 18–22].

Inappropriate use of antimicrobials and corticosteroids in the treatment of reproductive disorders or manipulation of obstetrical procedures like retained placenta and others in camels and cows favor bacterial contamination of vagina and the subsequent invasion of the uterine environment [23, 24]. Studies have shown that uterine infection is a significant cause of reproductive failure and infertility in both dromedary camelids [5] and cows [25]. On the other hand, in cows, bacterial pathogens are associated with delayed uterine involution and failure to conceive on one or more cycles in the same season [24, 26]. *Actinomyces* species, *Escherichia coli*, *Fusobacterium* species, *Pasteurella* species, *Pseudomonas* species and *Staphylococcus* species are the most common and economically important bacteria species associated with uterine infections in both animal species [26, 27].

Reproductive inefficiency of animals due to pathological lesions of female genitalia causes huge economic loss. To circumvent the problem, isolation of bacteria associated with uterine infection and histopathological investigations of lesions are critical for early diagnosis and management of poor reproductive performances. Most previously reported studies were based on gross pathological lesion observation and were involved either cows [7–9] or female dromedary camels [11]. Comparative studies on pathological lesions in reproductive tract and isolation and identification of bacteria involved in uterine disorder are very few in general and were not yet attempted in Ethiopia. To narrow these gaps, the current study was aimed to identify and compare the types and frequencies of reproductive organ pathological lesions between cows and female dromedary camels; describe and characterize gross and microscopic lesions of observed abnormalities. Additionally, the study also tried to isolate and identify aerobic bacteria associated with uterine lesions in both cows and female dromedary camels.

## Results

**Fig. 1:**
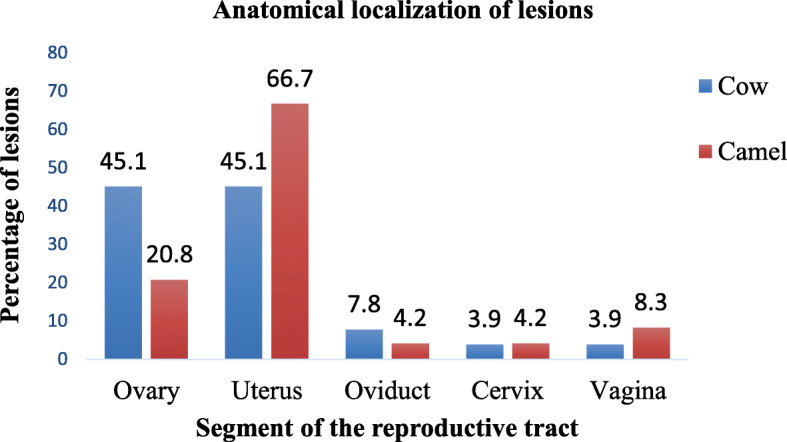
Anatomical localization of reproductive organ lesions in cow and dromedary camel

The detail of observed lesion types with frequencies was shown on Tables 1, 2 and 3 below. Of the total 280 reproductive tracts (140 for each group of cows and dromedaries), 48 (34.2%) and 51 (36.4%) diseased reproductive tracts were observed in female dromedary camels and cows, respectively. Regarding the relative occurrence of the reproductive tract lesions in each species, it was found a similar prevalence of lesions in the ovary/bursa and the uterus (45.1%, each) in cows, contrasting with the dromedary, which showed a higher prevalence of uterine than ovarian/bursal lesions (66.7% vs. 20.8%) (Fig. 1). Vaginal and oviductal lesions were the less prevalent lesions of the reproductive tract in both cows and dromedary females; nevertheless, an opposite prevalence pattern was observed with oviductal lesions observed more frequently in cows (Fig. 1). Overall the prevalence of follicular cyst was higher in animals with good body condition as compared to animals with medium or poor body condition (Table 1). Also, older animals in both species were more prone to chronic endometritis than other age groups (Table 2).

**Table 1:** Frequency (%) of ovarian lesions

Variables	Category	Follicular cyst	Luteal cyst	Paraovarian cyst	Inactive ovary	Ovarian-bursal adhesion	Oophoritis	Ovarian hydrobursitis
Species	Cow	6 (26.1)	3 (13)	2 (8.7)	4 (17.4)	7 (30.4)	1 (4.3)	0
	Camel	4 (40)	2 (20)	2 (20)	1 (10)	0	0	1 (10)
Age	Young	1 (20)	1 (20)	1 (20)	1 (20)	1 (20)	0	0
	Adult	7 (38.9)	2 (11.1)	3 (16.7)	3 (16.7)	3 (16.7)	0	0
	Old	2 (20)	2 (20)	0	1 (10)	3 (30)	1 (10)	1 (10)
BCS	Poor	1 (10)	2 (20)	0	4 (40)	2 (20)	0	1 (10)
	Medium	1 (14.3)	1 (14.3)	3 (42.9)	0	1 (14.3)	1 (14.3)	0
	Good	8 (50)	2 (12.5)	1 (6.3)	1 (6.3)	4 (25)	0	0

**Table 2:** Frequency (%) of uterine lesions

Variables	Category	Cervicitis	Acute Endometiritis	Chronic Endometiritis	Catarrhal Endometiritis	Suppurative Endometritis	Leiomyoma
Species	Cow	2 (8.7)	9 (39.1)	7 (30.4)	2 (8.7)	2 (8.7)	1 (4.4)
	Camel	2 (6.3)	12 (37.5)	16 (50)	2 (6.3)	0	0
Age	Young	0	2 (25)	5 (62.5)	1 (12.5)	0	0
	Adult	2 (1.52)	11 (61.1)	5 (27.8)	0	2 (11.1)	0
	Old	2 (1.67)	8 (32)	13 (52)	3 (12)	0	1 (4)
BCS	Poor	1 (1.13)	7 (35)	7 (35)	3 (15)	2 (10)	1 (5)
	Medium	1 (1.75	5 (50)	5 (50)	0	0	0
	Good	2 (1.48)	9 (42.9)	11 (52.4)	1 (4.8)	0	0

**Table 3:** Frequency (%) of oviductal and vaginal lesion

Variables	Category	Hemosalpinx	Pyosalpinx	Vaginitis	Vaginal myositis
Species	Cow	2 (50)	2 (50)	2 (100)	0
	Camel	1 (50)	1 (50)	1 (25)	3 (75)
Age	Young	0	0	0	0
	Adult	2 (1.52)	2 (1.52)	2 (1.52)	2 (1.52)
	Old	2 (1.66)	1 (0.83)	1 (0.83)	1 (0.83)
BCS	Poor	2 (2.27)	1 (1.13)	1 (1.13)	2 (2.27)
	Medium	0	1 (1.75)	1 (1.75)	0
	Good	2 (1.48)	1 (0.74)	1 (0.74)	1 (0.74)

### Ovarian and oviductal lesions

Ovarian abnormalities were observed in 23 (45.1%) and 10 (20.8%) of examined cows and female dromedary camels, respectively. Follicular cyst, luteal cyst, inactive ovary, paraovarian cyst, ovarian bursal adhesion, oopharitis and hydrobursitis were among the observed lesions (Table 1). Among these lesions, ovarian bursal adhesion and oopharitis were observed only in cows whereas hydrobursitis was seen only in dromedary camel.

**Fig. 2:**
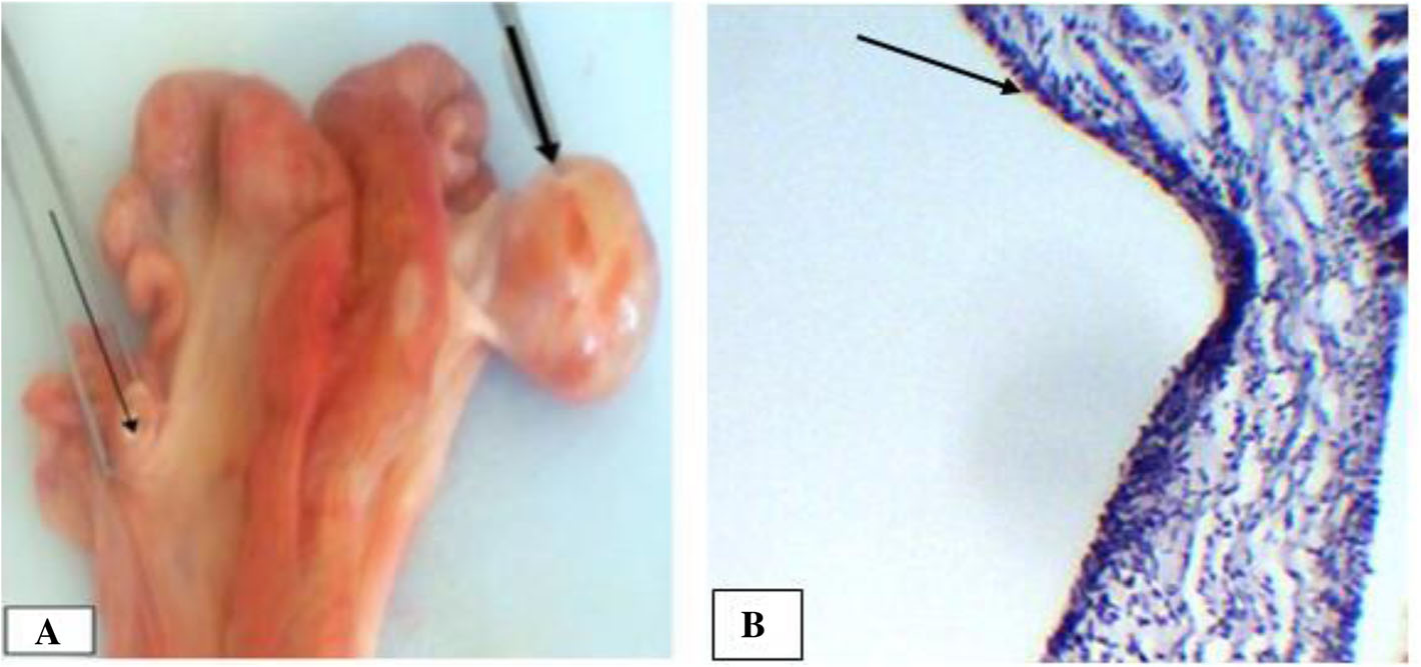
Follicular cysts in a cow. **a**. On gross inspection, it can be noticed that the cyst occupies the entire right ovary and very thin wall due to distention by follicular fluid indicated (short thick arrow); the contralateral ovary is inactive (arrow); **b**. Microscopic evaluation showed the cyst walls composed of extremely thin granulosa cells (black arrow). Hematoxylin-Eosin stain (400X- overall magnification)

Follicular cysts were seen in 26.1 and 40% of ovarian lesions detected in cows and dromedary camels, respectively. The lesions were unilateral in both species. In cows, a balanced distribution of the cysts between the two ovaries were observed, while in dromedary camel, a higher prevalence of ovarian cyst on left ovary was recorded (3 vs 1 in the right ovary). Macroscopically, most of the cysts were spherical in shape and occupied the ovarian cortex in both species. The cysts diameter ranged from 30 mm to 55 mm, thin walls and containing of slightly opaque and straw-colored serous fluid. One follicular cyst in a cow was accompanied by an ipsilateral inactive ovary (Fig. 2a) and endometritis. Microscopically, the follicular cysts were lined by few layers of granulosa cells, as large proportion of the granulosa cells were degenerated due to the pressure exerted by the follicular fluids. It was difficult to differentiate the theca interna from the externa as the cells were compressed by the fluid pressure (Fig. 2b). Moreover, larger cysts were also induced atrophy on the adjacent ovarian tissues and originated degeneration of the oocyte and the surrounding cells.

Among the observed ovarian lesions, luteal cysts were seen in 13 and 20% of examined cows and dromedary camels, respectively. In cows, two cysts were on the right ovary and one on the left while in dromedary camels, all cysts were observed on the right ovary. Macroscopically, the luteal cysts were thick walled, opaque and have meat like consistency. The wall of the cysts was lined by a whitish brown or yellow membrane. Furthermore, in cows, all the luteal cysts appeared as a single rounded mass on the surface of the ovary whereas in one camel multiple luteal cyst were observed. The diameter of the luteal cysts ranged from 20 to 25 mm and microscopically, the wall of the luteal cysts was formed from thick layers of lutein and granulosa cells rich in lipid content. They contained homogenous eosinophilic structure mixed with some luteal cells in the lumens.

Paraovarian cyst was observed in both dromedary camel and cows with a frequency of 20 and 8.7%, respectively. Macroscopically, the cysts were unilateral and on the left ovary in both species and located either in mesovarium or in mesosalpinx ligament. These cysts were appeared transparent containing clear watery fluid and having a diameter of 10–20 mm in diameter thin wall. Hydrobursitis was observed in 10% of examined dromedary camels but, not in cows. The size of the affected bursa was about 5 × 6.4 cm and was unilateral. Moreover, about 25 ml of watery and yellowish fluid was found accumulated in the bursa.

**Fig. 3:**
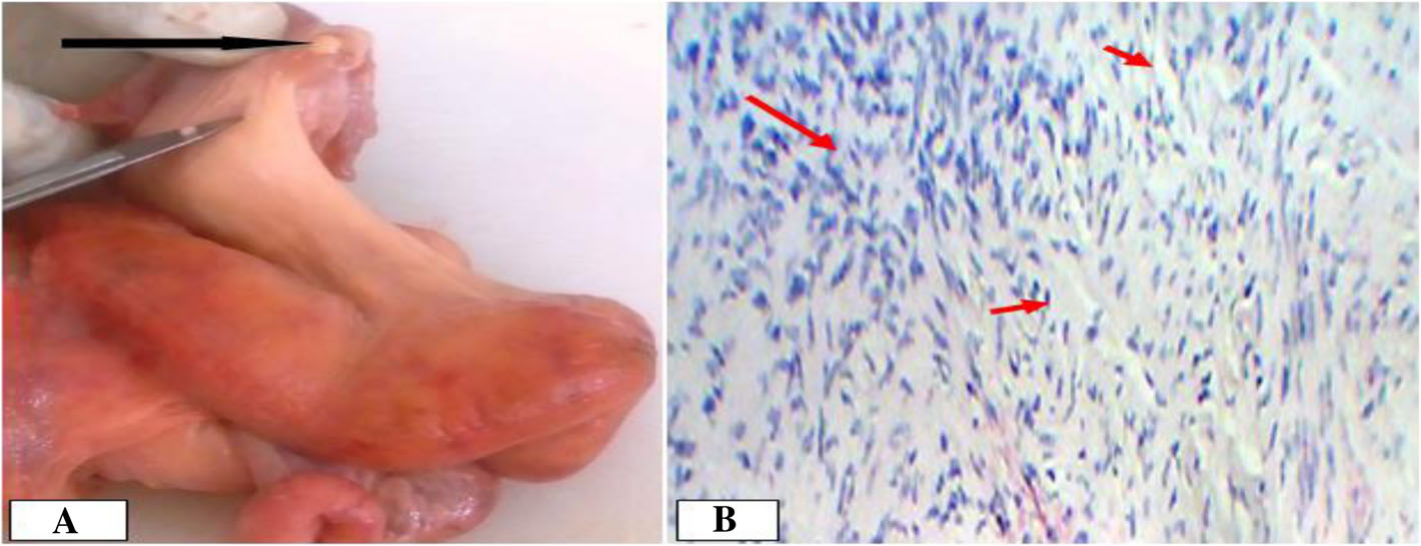
An inactive cow ovary. **a**. Note the small size ovary. **b**. Excessive fibrous connective tissues proliferation with complete absence of follicular or luteal development. Hematoxylin-Eosin stain (400X- overall magnification)

The frequency of occurrence of an inactive ovary was 17.4% in cows and 10% in dromedary camels. In dromedaries, it was generally a bilateral condition and the ovary was very small, oval in shape, and measuring 1 cm by 1 cm whereas in cows, an inactive ovary was always a unilateral condition; three of them on the right ovary and one on the left. The size of the affected ovary was varied from 1.6 cm by 1.1 cm to 1.5 cm by 1.0 cm. Macroscopically the ovaries were smaller in size, firmer in consistency and contained very small follicles on the surface. Microscopically, excessive fibrous connective tissue proliferation with complete absence of follicular or luteal developments was seen (Fig. 3 a and b).

**Fig. 4:**
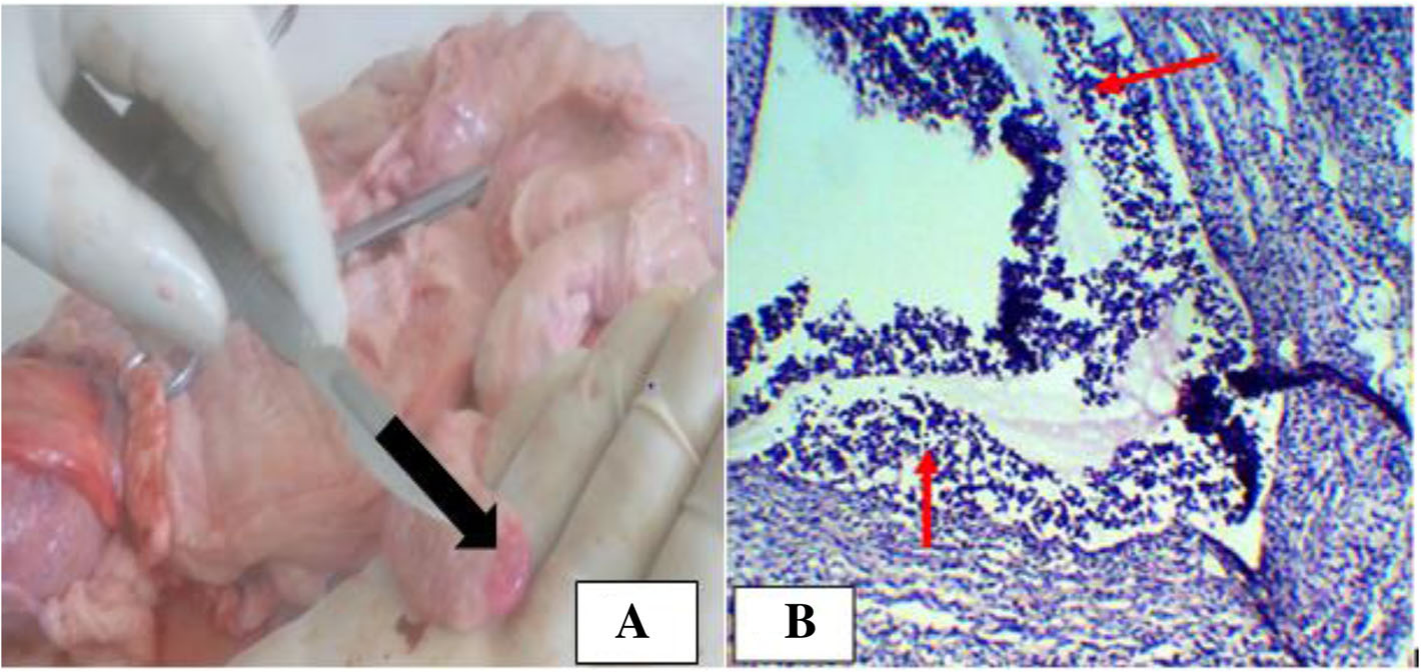
Oophoritis in a camel female. **a**. Note the hyperemic ovary; **b**. Inflammatory cells infiltrations in ovarian medullary region. Hematoxylin-Eosin stain (400X- overall magnification)

One case of oophoritis (4.3%) was observed in cow but was not in any of the dromedary camels. This lesion might be related to the existence of ovarian bursal adhesion as they co-existed. Macroscopically, the ovary was hyperemic and slightly swollen. Microscopically, the ovarian medulla was infiltrated by inflammatory cells (Fig. 4 a and b).

Ovariobursal adhesion was the most frequently observed (30.4%) ovarian lesions in cows, however, none was observed in dromedary camels. Except for a single adhesion which was bilateral, all examined lesions were unilateral. Four of the adhesions were on the right and two were on the left ovary. In one case, ovariobursal adhesion co-existed with oophoritis. Macroscopically, the ovaries were found adherent to the bursa and surrounded by a layer of connective tissue. The severity of adhesions was varied from case to case. In five cases the adhesions were mild with sparse strands of connective tissue between the ovary and bursa while in two cases the adhesions were so severe that the ovaries were completely encapsulated in thick fibrous connective tissues.

**Fig. 5:**
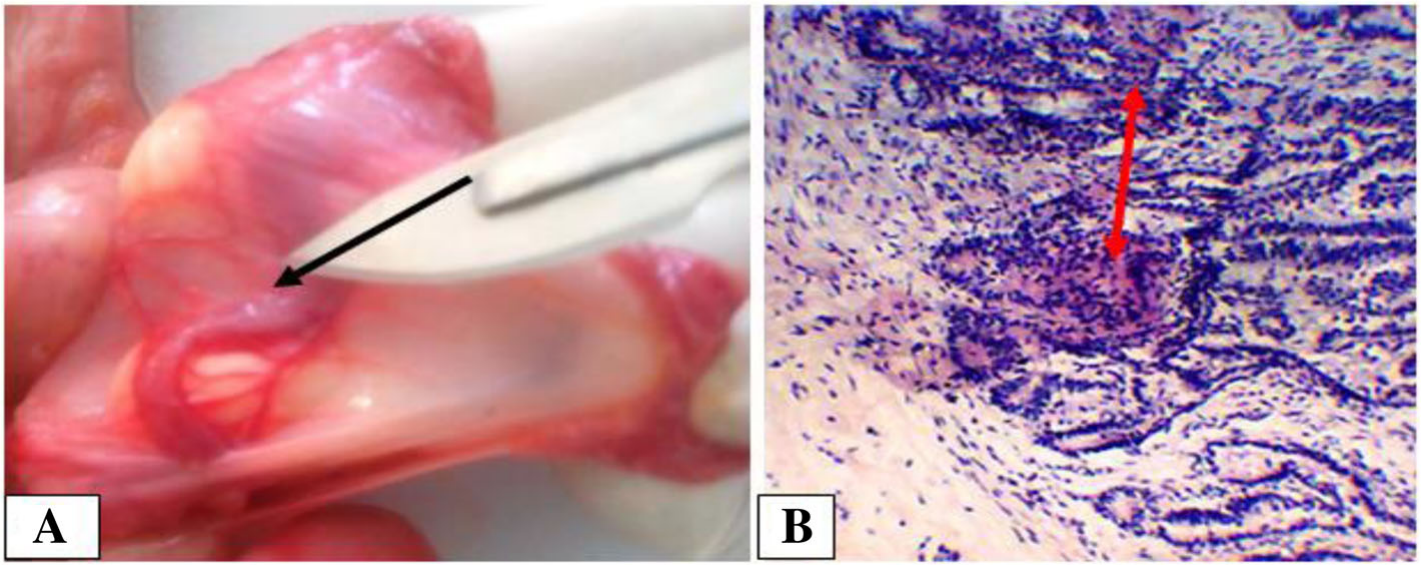
Hemosalphinx in a cow. **a**. Note the mucosa was hyperemic and the lumen filled with bloodish fluid; **b**. Microscopically edema and inflammatory cell infiltrations (red arrow). Hematoxylin-Eosin stain (400X- overall magnification)

Hemosalpinx was observed in one dromedary camel and three cows. In the dromedary camel, it was unilateral and observed in the right oviduct. However, it was bilateral in one cow and unilateral in two cows and seen in the right oviduct. Macroscopically, in both species the oviduct was slightly enlarged in size (Fig. 5a) and the mucosa was hyperemic and filled with blood. Microscopically, hyperplasia of the lining epithelium and congestion of blood vessels with inflammatory cellular infiltration were noticed (Fig. 5b). Pyosalpinx was detected in one camel and two cows. Macroscopically, the affection was bilateral in both species. Moreover, the oviduct was enlarged and distended with pus. In one cow, pyosalpinx was accompanied with suppurative endometritis.

### Uterine lesions

Uterine lesions were more frequently observed in dromedary camels (66.7%) than in cows (45.1%). Except for a leiomyoma, all uterine lesions were microscopically confirmed as inflammatory in both species. Acute, chronic and catarrhal endometritis were commonly observed in either species, however, suppurative endometritis was observed only in cows (Table 2).

**Fig. 6:**
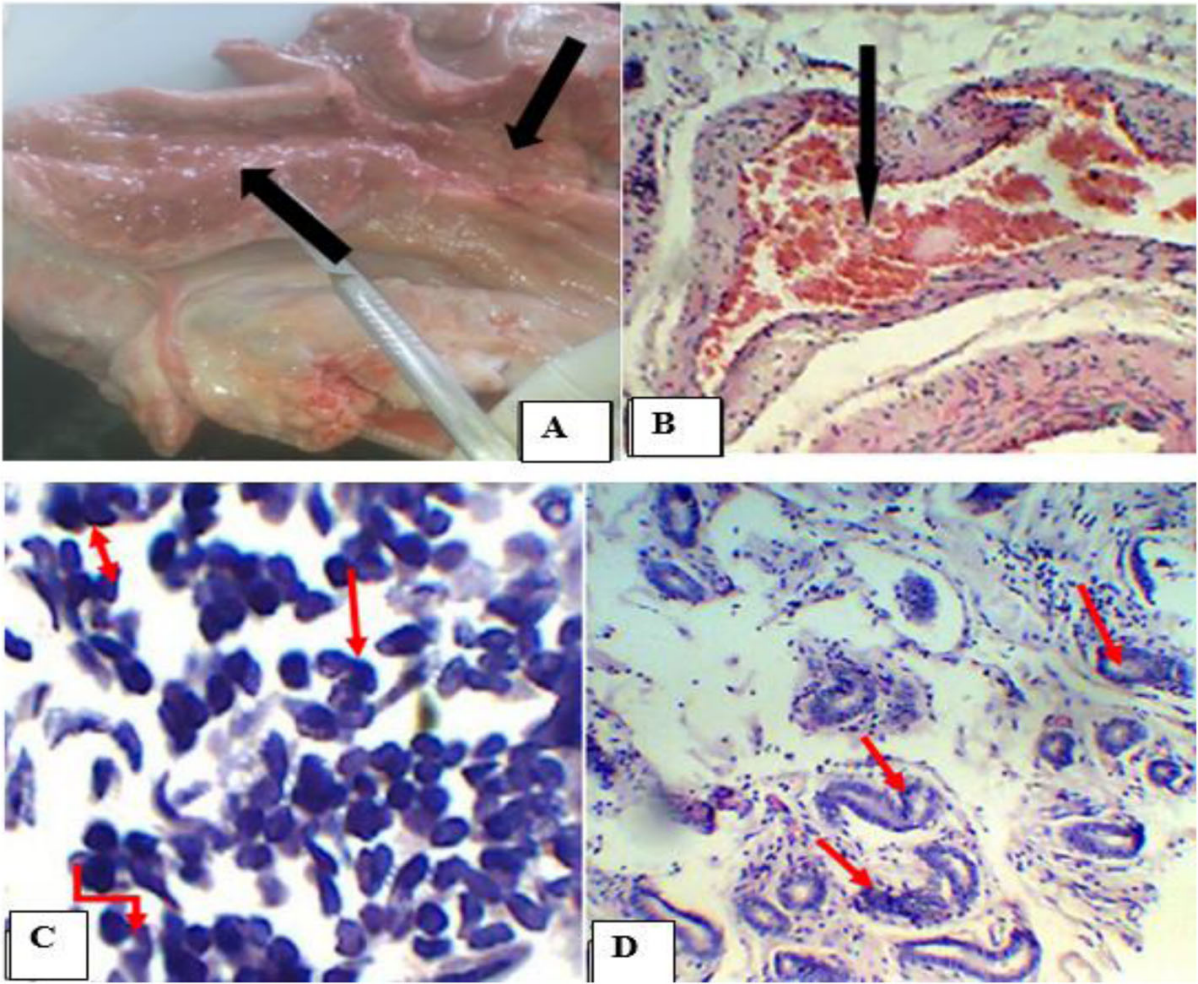
Endometritis in camels. **a**. Severely hyperemic acute endometritis with blood tinged exudates in the upper corner; **b**. Congestion of endometrial blood vessels; **c**. Aggregation of neutrophyls. Hematoxylin-Eosin stain (100X- overall magnification); **d**. Misshapen and atrophied endometrial gland. Hematoxylin-Eosin stain (400X- overall magnification)

Acute endometritis was more frequently recorded in cows (39.1%) than dromedary camels (37.5%) (Table 2) and it was accompanied by luteal cyst in two of the dromedary camels. Macroscopically, the affected uteri were enlarged and the mucosa was either severely congested (red brown) or severely reddened (Fig. 6). Furthermore, in three cases of dromedary camel and one case in cows, thick blood tinged exudates were seen in the uterine lumen. Microscopically, necrosis or sloughing of epithelia were found in most cases, along with congestion or hyperemia of endometrial blood vessels, especially, in basilar endometrial region. Moreover, polymorphonuclear cells, mostly neutrophils, were infiltrating the endometrium. Excessive peri-glandular cuffing of cells and atrophy of endometrial glands were characteristics of acute endometritis.

**Fig. 7:**
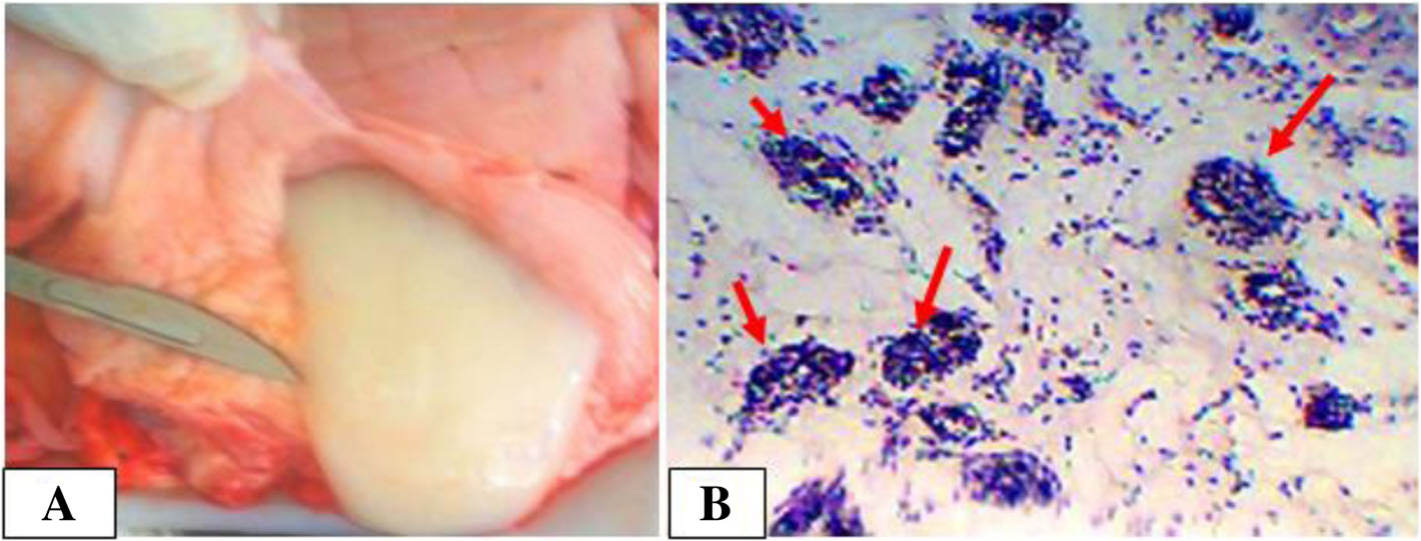
Catarrhal endometritis in a cow. **a**. Note the catarrhal exudates discharged from vagina; **b**. Microscopic endometrial gland degeneration. Hematoxylin-Eosin stain (400X- overall magnification)

Suppurative endometritis were observed only in cows with a frequency of 8.7%. Macroscopically, the endometrium was congested and covered with thick creamy white pus (Fig. 7a). The lesions were further expanded to uterine horn but, no purulent material was observed in uterine horn. Microscopically, the lesion was characterized by infiltration of neutrophils into the endometrium with severe congestion of blood vessels and distortion of some endometrial glands. Catarrhal endometritis was observed in two dromedary camels and two cows. Macroscopically, the uterus was enlarged with slightly congested, edematous mucosa which was covered by thick catarrhal exudates. In one case of dromedary camel, the exudate was further expanded to cervix and vaginal mucosa with outward discharge. Microscopically, there was congestion of endometrial blood vessels, lymphocyte infiltration in the mucosa and sub-mucosa with alternative areas of epithelial desquamation and hyperplasia of lining epithelium. Degeneration of the endometrial glands were also observed (Fig. 7b).

**Fig. 8:**
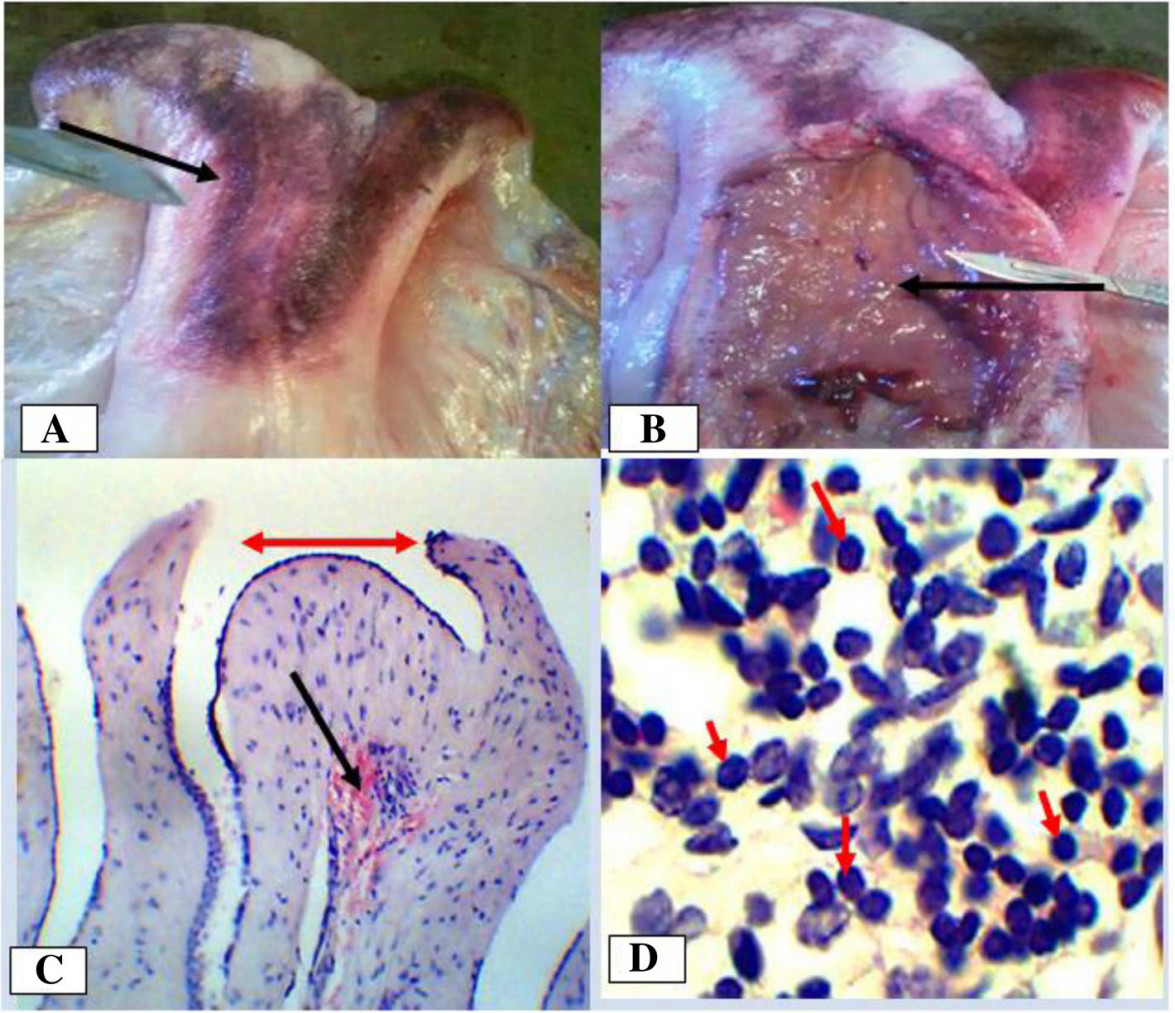
Chronic endometritis in a camelid female. **a**. Note severe congestion visible from outside; **b**. Severely congested and thickened mucosa on incision; **c**. Severely hyperplastic mesetholium (red two-sided arrow) and hemorrhage (black arrow) Hematoxylin-Eosin stain (400X- overall magnification); **d**. Lymphocytes infiltrations in endometrium. Hematoxylin-Eosin stain (100X- overall magnification)

Chronic endometritis was more frequent in dromedary camels (50%) than cows (30.4%) (Table 2). In one case in cows, the lesion existed concomitantly with a luteal cyst, vaginitis and cervicitis. Macroscopically, most of the uteri were thick, doughy, rigid, and their mucosa was severely congested. In one case in dromedary camels, the uterus was severely congested, with corrugation of the perimetrium and dark brown hemorrhage on the external surface (Fig. 8 a and b). Microscopically, the uterine mesothelium was hyperplastic with polypoid like projections and diffuse thickening of basal fibrous connective tissue. Most affected uteri were characterized by endometrial glandular degeneration, a mononuclear inflammatory infiltrate containing mostly lymphocytes, macrophages and to a lesser extent neutrophil. In some cases, hyperplasia of endometrial epithelium was observed (Fig. 8 c and d).

**Fig. 9:**
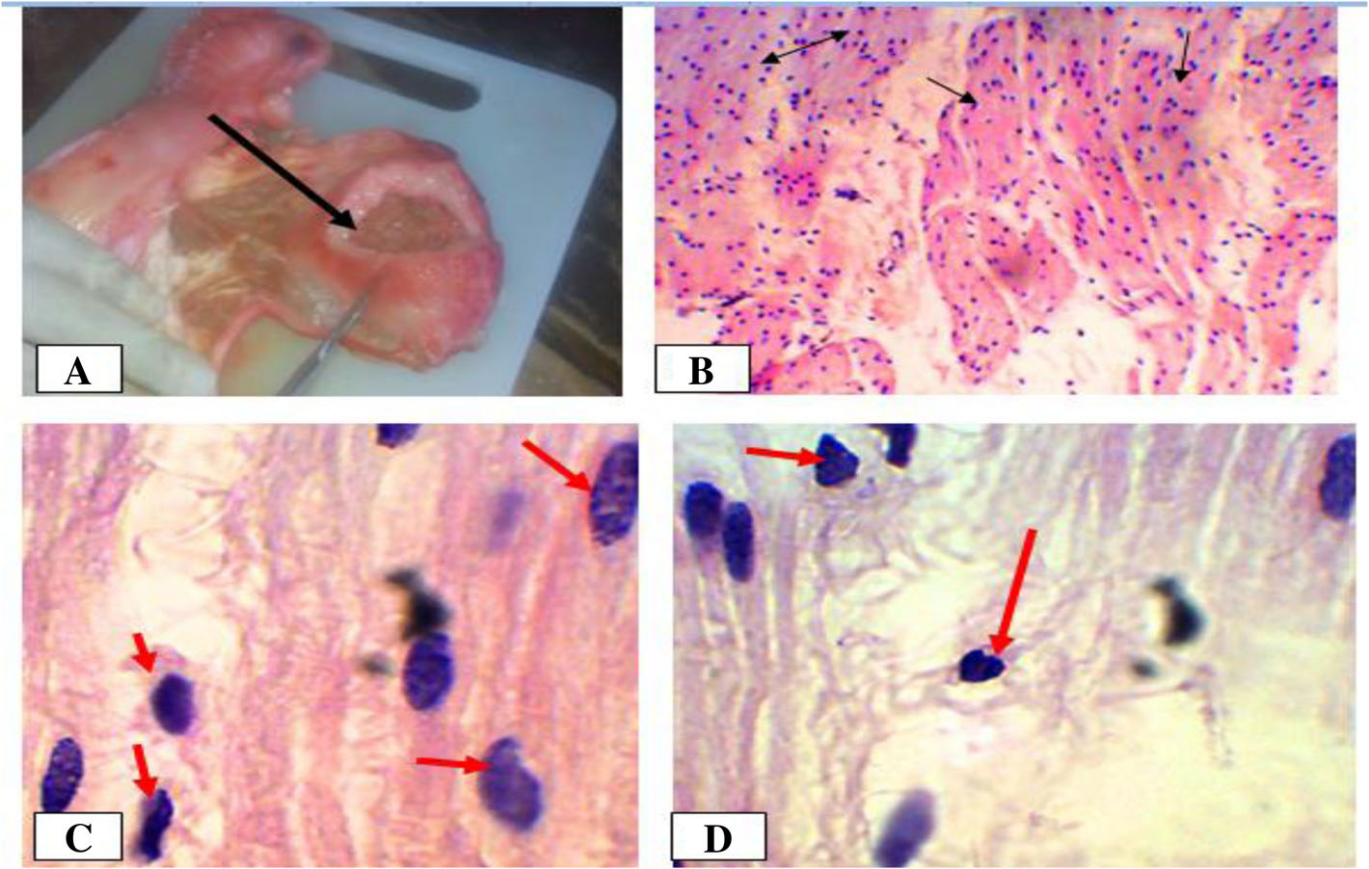
Leiomyoma in a cow. **a**. Firmly attached mass originated from body of uterus; **b**. Bundles of smooth muscles running in various directions and interlaced with each other are observed. Hematoxylin-Eosin stain (400X- overall magnification); **c**. variation of nucleus shape; **d**. slight mitotic figure (red arrow). Hematoxylin-Eosin stain (100X- overall magnification)

Only one uterine tumor was observed in a cow and was diagnosed as uterine leiomyoma whereas none was observed in dromedary camels. Macroscopically, the neoplasm was well-circumscribed, firmly attached on the body of the uterus with a diameter of 10 cm and a whorl-like and trabeculated deposition on cut surface. Microscopically, the leiomyoma was comprised of smooth muscle cells and connective tissue components. Largely, composed of interlacing (interweaving) bundles of smooth muscle fibers with acidophilic cytoplasm and elongated and rounded blunt ending nuclei. The fibers were usually fusiform or stellate in shape, possessed large, ovoid to elongated nuclei and sometimes multiple nucleoli. Slight pleomorphism and little mitotic activity was also observed (Fig. 9).

### Cervico-vaginal lesions

Cervicitis was recorded in both species of animals with a frequency of 4.2% in dromedary camels and 3.9% in cows. In general, cervical lesions were observed with low incidence than the rest of reproductive organ lesions in both species (Table 3). In both species, the observed cervicitis was always associated with endometritis. Macroscopically, the cervix was slightly enlarged with congested and edematous mucosa and covered with whitish viscous exudates.

**Fig. 10:**
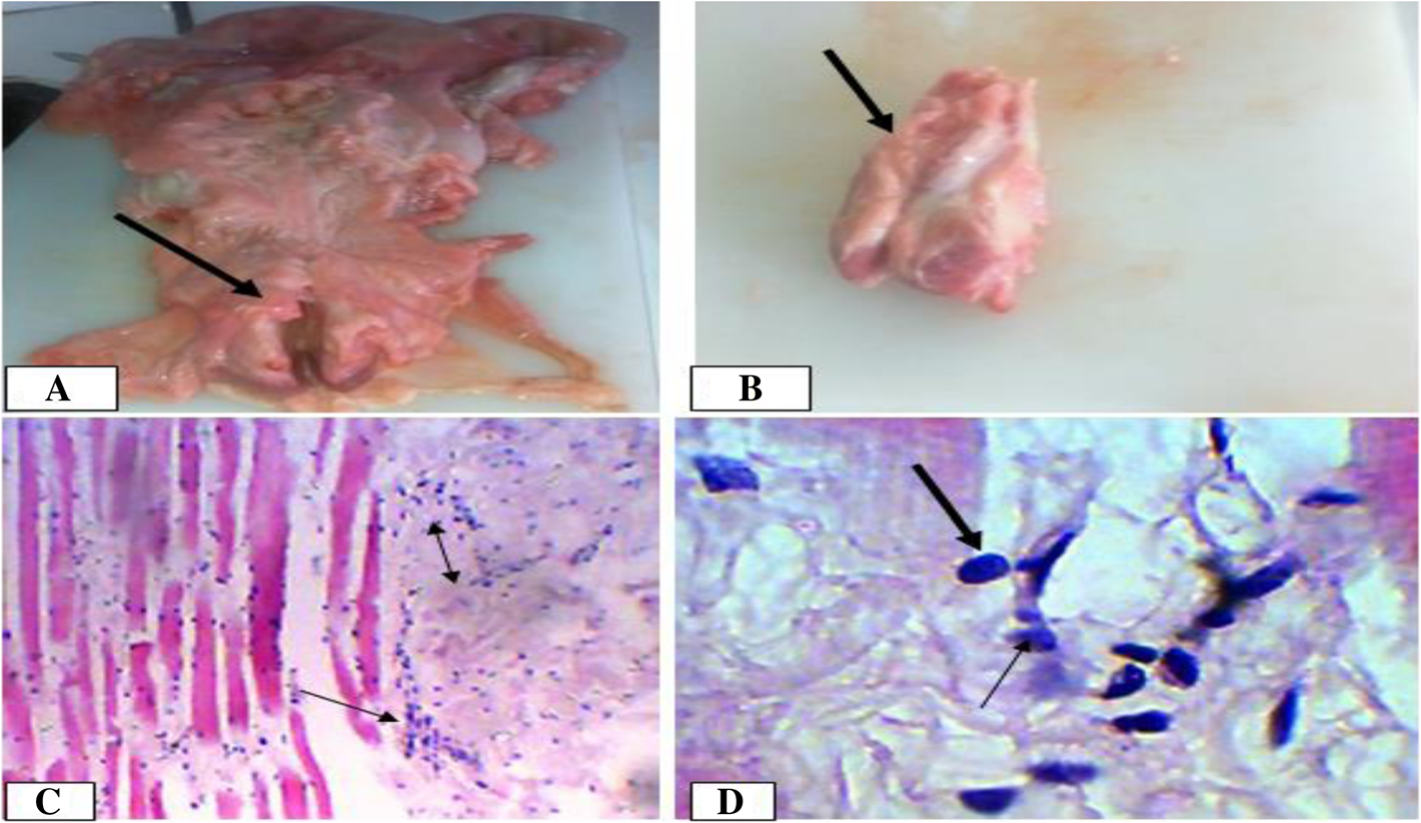
Vaginal myositis in a camel female. **a**. Cross section of swelling mass in the wall of the vagina; **b**. Tumor like mass after removed from the wall; **c**. Sloughed myocytes Hematoxylin-Eosin stain (400X- overall magnification); **d**. lymphocytes infiltration between myocytes. Hematoxylin-Eosin stain (100X- overall magnification)

Generally, vaginal lesions were more frequently observed in dromedary camels (8.3%) than cows (3.9%). Vaginitis and vaginal lymphocytic myositis were the kinds of vaginal lesions observed in dromedary camels with a frequency of 25 and 75%, respectively. In cows, however, only vaginitis was observed (Table 3). Macroscopically, the mucosa appeared slightly swollen and showed diffuse, non-homogeneous congestion and hyperemia. Moreover, in one of the three cases in camels in which vaginal lymphocytic myositis was observed, the lesion was consistent with chronic endometritis. Macroscopically, in all vaginal lymphocytic myositis cases, the lesion appeared as single, large sized (7–10 cm in diameter) mass on the lateral wall of the vagina just cranial to vulvar commissure and was soft upon palpation. Microscopically, these lesions were characterized by huge lymphocyte infiltration into the smooth muscle fibers and some muscular degeneration. In some regions, the muscle fibers were necrotized and connective tissues were proliferated (Fig. 10).

### Bacterial isolates from the uterine lesions of cows and camels

**Fig. 11:**
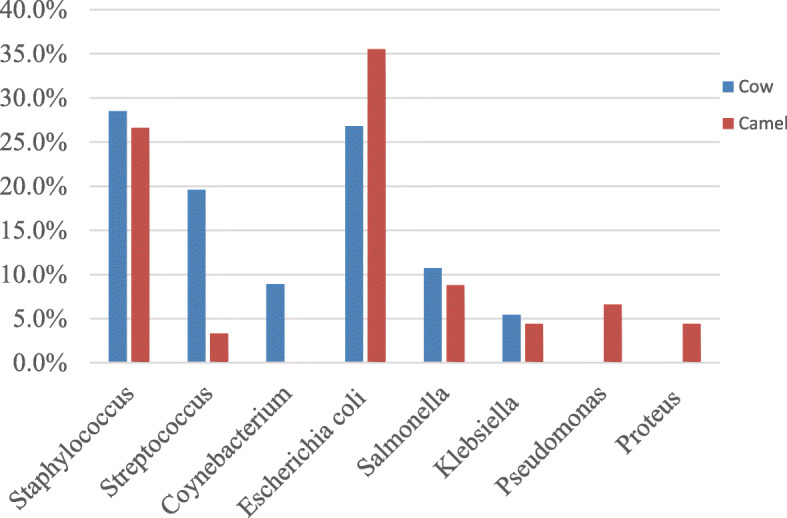
Graphical presentation of bacteria isolated from uterine lesions

Fifty uterine tissue samples (30 from camels and 20 from cows) with acute, chronic, catarrhal and suppurative endometritis were cultured aerobically for bacterial isolation, 48 of which were found to be positive. A total of 101 isolates were recovered (56 from cows and 45 from dromedary camels) and majority of the isolates were presented as a mix bacterial population. Among bacterial pathogens isolated from cows, prevalence was distributed as follows: *Staphylococcus* spp. (28.5%), *Escherichia coli* (26.8%), *Streptococci* spp. (19.6%), *Salmonella* spp. (10.7%), *Coynebacterium* spp. (8.9%) and *Klebsiella* spp. (5.4%). In dromedary camels, the most common isolates included *E. coli* (35.5%), *Staphylococcus* spp. (26.6%), *Salmonella* spp. (8.8%), *Pseudomonas* spp. (6.6%), *Proteus* spp. and *Klebsiella* spp. (4.4%), and *Streptococcus* spp. (3.3%) (Fig. 11). The common bacterial isolates identified from both species (*Staphylococcus* spp., *Streptococcus* spp., *Escherichia coli*, *Salmonella* spp. and *Klebsiella* spp) accounted for more than 90% of isolated bacteria. Moreover, *Pseudomonas* spp. and *Proteus* spp. were only isolated from camel uteri whereas *Corynebacterium* spp. was only isolated from cows’ uteri.

## Discussion

Reproductive abnormalities play an important role in animal breeding either by causing infertility or sterility, and thus inflict heavy economic losses to the livestock owners. Animals with reproductive problems and low milk production are usually culled. For minimization of these losses, important disorders of genital organs and their incidence must be defined [28]. Although previous reports claimed that camels are resistant to various disease conditions [29], the current study suggests that most reproductive disorders occurred similarly in both species of animals. This is in agreement with previous study reports [30, 31] documenting that camels to be quite as susceptible as other livestock species to various disease conditions.

In this study the prevalence of uterine lesion was greater in dromedary camels than in cows, even if a previous report showed an opposite trend [27]. This variation might be attributed to differences in season, management, geographical environment, level of nutrition and health management of animals. In the current study endometritis was the major uterine lesion observed in dromedary camels. This could be attributed to different factors like repeated insults of the uterus due to improper mating practices [32], postpartum complications or unsanitary gynecological manipulations [5]. Moreover, in line with the above statement, almost all of the examined animals in this study were culled because of reproductive problems and were no more productive.

Occurrences of chronic endometritis in this study varied within age groups in both dromedary camels and cows. Old dromedary camels and cows were more prone to chronic endometritis than other age groups of animals which was in accordance with a report by Waheed et al. [33]. This might be associated to the fact that older animals are associated with increased frequency of mating, parturitions and/or repeated postpartum complications than other age groups. Agreeing with the current study, previous reports exists on uterine leiomyoma in cows [34–36], and. Although the etiology of the uterine leiomyoma is unclear [34], in this study it was found that the leiomyoma was composed of smooth muscle neoplastic cells accompanied by varying quantities of non-glandular connective tissue. This was in agreement with the report of Kennedy and Miller [37].

Even though no cases of leiomyoma were observed in camels in this study, previous studies indicated that female dromedary camels may also develop leiomyoma [10]. The low frequency of cervical lesions observed in this study may be due to good defense action of the mucous secreting epithelium of the cervix against bacterial invasion [38]. The observed cervicitis in the present study were associated with uterine affection or inflammation of endometrium and this was in line with the report by Shawky et al. [10].

The prevalence of ovarian lesions of cows in this study were almost twice those recorded in dromedary camels. This might be attributed to the high production potential of the Holstein Friesian breeds of cows which were highly selected for milk yield. According to the reports of Opsomer et al. [39] and Butler [40], dairy cows experience negative energy balance especially at early lactation and with higher milk yield, and are considered to be at risk of developing ovarian disorders. As to our knowledge, most dairy cows in Ethiopia were owned by small holder farmers with poor management practices in which exposure to different reproductive problems is inevitable.

The present study showed higher prevalence of follicular cysts in dromedary camels as compared to cows. This might be associated to the fact that dromedary camels ordinarily are liable to develop follicular ovarian cysts in the absence of coitus [41]. Additionally, this can also be associated to various influencing factors like level of milk production; feeding, management and exercise which might affect the prevalence of cystic follicle in either of the animal species [42]. Though it is difficult to determine the exact causes of ovarian follicular cyst, it can be realized that it develops when one or more follicles fail to ovulate and subsequently fail to regress maintaining growth and steroidogenesis [43, 44].

In this study the prevalence of follicular cyst in both species of animals was found to vary according to body condition score, the occurrence of follicular cysts being higher in animals with good body condition. This was in agreement with earlier reports by Tibary and Anouassi [23] and Abalti et al. [7] in which camels and zebu cattle with ovarian cysts had a general body condition fair to good. Furthermore, it is found documented elsewhere that dairy animals that were exposed to negative energy balance especially at early lactation and with higher milk yield were considered to be at risk of developing ovarian cysts [39, 40]. Microscopically, degeneration of surrounding theca and granulosa cells were seen in both species which was comparable with various previous reports [10, 11, 20, 28].

The current study showed that the prevalence of luteal cyst was lower than that of the follicular cyst in both species of animals. This might be due to the fact that luteal cysts originate from luteinization of follicular cyst, which results from luteinization of granulosa cells in the absence of ovulation [10]. Moreover, they are often considered to be a later form of ovarian follicular cysts and therefore the causes pertaining to follicular cysts can also be considered the original causes of luteal cysts [44]. The macroscopic and microscopic findings of the present study was in line with previous study reports [10, 20].

In this study ovarian bursal adhesion was only seen in cows. Although there was no history of pregnancy complications in the current study, previous study reports indicated that extreme adhesions have probably resulted from pregnancy complications like retained fetal membrane and endometritis [45]. Furthermore, this lesion can also result from hemorrhage due to harsh manipulation of the ovaries or attempts to rupture an ovulatory follicle or from oophoritis, ovarian hydrobursitis and peritonitis [23]. The prevalence of ovarian bursal adhesion observed in this study was in agreement with previous reports [7, 9, 17]. However, it was higher than that reported by Hatipoglu et al. [28]. This variation might be attributed to differences in breed, management and level of nutrition.

In this study oophoritis was observed only in cows and was in line with the report of camel oophoritis by Mahmoud et al. [20]. As it is documented by Fathalla et al. [46], oophoritis seems to be a rare pathological condition of bovine ovary while peri-oophoritis is commonly found. The prevalence of hydrobursitis in camel in this study was approximately similar to that of Al-Afaleq et al. [19] and lower than the report of Ali et al. [18] and Mohammed et al. [6]. The macroscopic findings of this lesion were in line with that of Mohammed et al. [6]. Available sources indicated that the incidence of hydrobursitis is relatively higher in animals with a background of reproductive failure which is also true in our case in which almost all of the slaughtered animals were with a history of reproductive failure [47].

In this study para-ovarian cysts were observed more in camels as compared to cows. These cysts are suspected to arise from persistent embryonic structures representing the vestiges of wolfian ducts [20]. In cows, these cysts are considered to be not interfere with the reproductive performance of the animal unless it leads to compression of the lumen of the oviduct [48, 49]. The reason why inactive ovary was observed with low frequency in dromedary camels than cows in this study might be related to seasonal breeding behavior of the camelids and the fact that current study was carried out during which most camels were cyclic. It was also justified that peak sexual activity of dromedary camel ranges from November to February [50]. Moreover, complete absence of follicular or luteal development and excessive fibrous connective tissue proliferation was observed in this study and was in agreement with previous reports [10, 11].

The frequency of oviduct lesions in the present study was low in both species and hemosalpinx was observed in both animal species which was in agreement with previous reports [26, 51]. Pyosalpinx accompanying suppurative endometritis observed in this study was in agreement with the report by Kennedy and Miller [37] who associated pyosalpinx with ascending infections. Moreover, Tibary and Anouassi [52] considered that untreated uterine infections can drive irreversible changes in oviducts thus resulting in sterility due to occlusion.

The observed pathological changes of vagina in both cows and dromedary camels herein were less frequent compared to that of uterine and ovarian lesions. This might be attributed to various factors of which the protective effect of stratified squamous epithelium of vaginal mucosa which proliferates and matures under the influence of estrogen and become more resistant to infection is of great concern. Furthermore, local production of lactic acid and its deposition into the epithelium is also considered [38]. The relatively higher frequency of vaginal lesions seen in female dromedary camels than cows might be related to traumatic injury during coitus [52]. Furthermore, Ali et al. [5] justified that the ethno-veterinary practices by herdsmen using unusual substances, like dates, black seeds, and salts might be irritant to the mucus membrane and leads to vaginal lesions in dromedary camels.

The current study also tried to isolate possible bacterial species from the affected uterus of both animal species. It was observed that the bacteria isolated from dromedary camels’ uteri were similar to those in cows. This might be associated with the camels husbandry practices in many parts of camel producing areas in Ethiopia and the rest of the world, which allow camels to graze together with other ruminants and mingling with them at watering points or market places, thereby creating conducive environment facilitating transmission of infectious pathogens circulating among other livestock species cohabiting within the same ecologic zone [53–55]. The bacteria isolated from endometritis might be suspected as the main causes of endometritis in both species. This finding was in support of the report by Tibary [56] that reported resistance of the uterus to infection and its ability to rid itself of microorganisms was diminished in the presence of degenerative changes in the endometrium.

The most common bacterial species isolated from cows’ and dromedary camels’ uterus with endometritis were *Staphylococcus*, *Streptococcus*, *Salmonella* and *Klebsiella* species and *Escherichia coli*. This finding was in line with previous study report by Mshelia et al. [31]. Most of the aforementioned bacterial species were attributed to the majority of clinical reproductive disorders [57]. *Proteus* and *Pseudomonas* species were isolated only from uterine lesions of dromedary camels and was in agreement with previous reports by Simenew et al. [11] and Al-Afaleq et al. [19].

## Conclusions

This study revealed one or more pathological abnormalities in reproductive organs of dromedary camels and cows slaughtered at Addis Ababa and Adama Municipal abattoirs and Akaki slaughterhouse. Uterine lesions were the major pathological disorders observed in dromedary camels while ovarian lesions were the most frequent in cows. Important bacterial species which considered to be important causes of uterine disorders were isolated from uterine lesions of both animal species examined. These observed bacterial isolates have to be investigated in susceptible female animals during the evaluation of animal’s pre-breeding status. Also, most of the pathological reproductive abnormalities detected in this study might be the cause of infertility in these animals and therefore the major reason for culling. Thus, in order to improve production outcomes, it is important to submit female animals to a breeding evaluation that should include bacteriological and histological evaluation before the breeding season, particularly if infertility is suspected. Besides, studies involving wider number of abattoirs and animals and correlation between individual’s reproductive lesions and hormonal disorders should be conducted to add with more figurative results. Furthermore, there is a need for panoptic studies basing uterine cytobrush and uterine biopsy to correlate bacterial isolates with uterine lesions. Additionally, trainings for concerned veterinarians, technicians and animal owners on reproductive health and management for proper intervention mechanisms should be implemented.

## Methods

### Study design, study area and study population

A cross-sectional study was conducted from November 2016 to May 2017 in Addis Ababa and Adama Municipal abattoirs, and Addis Ababa Akaki Kality camel slaughterhouse. Addis Ababa municipal abattoir is located at the central part of Addis Ababa whereas Akaki camel slaughterhouse is located in the south-eastern outskirt of Addis Ababa at 20 Km away from the center of Addis Ababa. Adama municipal abattoir is located in Adama town of Oromia regional state at about 99 Km away from the center of the capital Addis Ababa.

The study population were female dromedary camels slaughtered at Addis Ababa, Akaki camel slaughterhouse and cows slaughtered at both Addis Ababa and Adama Municipal abattoirs. All animals included in this study were culled due to either aging or possessing a history of reproductive failure (e.g., repeated breeding, anestrous, abortion, chronic mastitis). In these abattoirs only few or even no cows were slaughtered per day which was also true for female dromedary camels. Taking this in to consideration, the current study was purposively targeted and included all cows and female dromedary camels slaughtered at both municipal abattoirs and Akaki camel slaughterhouse, respectively during the study period. For sample collection, abattoirs were visited once every week for a total of 28 weeks for both animal species to provide sufficient time for sample processing at the laboratory. In both cases, on average, three to seven animals were slaughtered in each abattoir and all slaughtered cows and female dromedary camels were considered for the study. Accordingly, a total of 280 animals (140 female dromedary camels and 140 cows) were slaughtered and examined during the study period for which tissue samples with grossly visible lesions were collected for histopathological and bacteriological examinations. Holstein-Friesian, crossed breed (mixed breed of local Borena and Holstein-Friesian) and local (Borena, Zebu, Arsi-Bale and Harar) breeds of cows were included in to the study.

Age, body condition score and origin of animals were gathered during ante mortem examination. Age of female dromedary camels was estimated by dental examination on the basis of their dental formulas and tartar deposition on the teeth as described in Mohammed et al. [6]. However, cows’ age was estimated according to Puck and Soliame [58]. Accordingly, age of animals was categorized as < 5 years as young, 6–11 years as adult and > 11 years as old in both species of animals. In Ethiopia, pre-pubertal animals are unfit for slaughter due to religious concern and hence only mature or post- pubertal animals were evaluated in this study. The animals’ body condition score (BCS) was evaluated on a scale of 0 to 5 by considering visual examination and fat cover palpation over the animal body following the description by [59]. Therefore, animals were categorized as poor for ≤2, as medium for ≤3 and as good for ≥4 of measurement scale.

### Sample collection and processing

During postmortem examination the entire reproductive tract was carefully removed intact from the pelvic cavity within 10–20 min of slaughter, placed on a sterile tray and taken to one corner of the abattoir, where they were visually examined and thoroughly palpated. A total of 280 reproductive tracts (140 from cows and 140 from dromedary camels) were obtained and evaluated during this study period. Each reproductive tract was opened along its longitudinal axis starting from the vagina down to the horns using sterile scissors and was observed for any morphological abnormality, color, odor and consistency [9, 60]. Obvious gross lesions were noted based on their appearance, type, location and frequency of occurrence as previously described by Jenberie et al. [61]. Tissues with grossly visible lesions were sampled for histopathological and bacteriological examinations. In both animal species samples for bacteriological examination were collected only from affected uterus. For histopathology, a tissue cut of 1–2 cm from the margins of apparently normal and affected parts were collected and fixed in 10% buffered formalin according to Talukder [62] and transported to National Animal Health Diagnostic Investigation Centre for histopathological processing.

For bacteriological examination, the surface of the uterus with lesion was decontaminated by a flame and tissue fragments from active lesions at the boundary were collected aseptically using sterile forceps, scissors and scalpel blade and placed into screw capped universal bottles containing sterile saline water [63]. Additionally, a sterile cotton swab was used to collect drag swab samples from the active lesions of uterine surface right after visual examination and prior to tissue sample collection. The swab was placed in a 50 ml falcon tube containing sterile peptone water for transportation. All the collected samples were labelled, tightly closed and placed in a cool box containing ice packs and transported to the veterinary microbiology laboratory of Addis Ababa University College of Veterinary Medicine and Agriculture for culturing within 1–2 h of collection. All bacteriological processes were conducted within 6-8 h of sample collection.

All laboratory bacteriological procedures were performed within 6–10 h of sample collection and processed according to previously established protocols [63]. Briefly, tissue samples were cut into pieces and inoculated in to Brain Heart infusion broth and aerobically incubated at 37 °C for 24 h. The same broth was also inoculated directly with the collected swabs and aerobically incubated at 37 °C for 24 h. Tubes were then observed after 24 h of incubation for growth (turbidity) and a loop-full of the suspected culture was streaked parallel on Sheep Blood agar (7%) and MacConkey agar and incubated aerobically at 37 °C for another 24 h. The blood agar plates were checked for presence of growth, hemolysis (types), colony morphology, size and shape. MacConkey agar plates were also checked for presence of growth, lactose fermentation and for colony morphology, size and shape. For primary identification, gram stain, catalase, oxidase and motility tests were conducted. Selective and differential media such as Mannitol salt agar, Edwards medium, Eosine methylene blue and Salmonella shigella agar were used for the suspected samples from the primary test results. After 24 h of incubation, the characteristic growth on selective medium was registered after which a colony was then further inoculated in to nutrient broth for further biochemical tests. In general, coagulase, indole, Methyl red (MR), Vogues-Prousker (VP), Citrate, Urease, Lysine and Triple Sugar Iron (TSI) tests were performed as secondary biochemical tests.

For histopathological examination, tissue specimens were fixed in buffered formalin and routinely processed for paraffin-embedding and hematoxylin-eosin staining in 4 to 5 μm sections [62]. Stained slides were examined under the microscope (at 10x, 40x and 100x magnification) using a phase contrast microscope (Nikon, Japan), and photomicrographs were taken.

### Data analysis

A database was created on spreadsheet of Microsoft Excel, 2010 and STATA version 13 statistical software was used for descriptive analysis. Descriptive summary statistics (frequencies and cross-tabulation) were computed for pathological lesions and uterine bacterial isolates prevalence. Uterine bacterial isolates were compared between species using descriptive statistics. Gross and histopathological lesions and findings were described qualitatively.

## Supplementary Information

**Additional file 1: **Major reproductive lesions and their macroscopic and microscopic characterization.

## Data Availability

All datasets generated and/or analyzed during the current study and supporting the findings of this study are not publicly available due to the fact that this study was part of the ongoing thematic research funded by Addis Ababa University, Research and Technology Transfer Office, but are available from the corresponding author at any time on reasonable request.

## References

[1] Arata MA (2015). A gross morphological study of genital organs from female zebu cattle in and around Jimma town (south-West Ethiopia). Int J Agro Vet and Medi Sci.

[2] Khaton R, Sarder M, Gofur M (2015). Biometrical studies of reproductive organs of dairy cows of different genotypes in Bangladesh. Asian J Ani Sci.

[3] Siddiqui HU, Ahmad A, Khan MZ (2005). Biometrical studies of testes of ram. J Agri Soci Sci.

[4] Khanvilkar AV, Samant SR, Ambore BN (2009). Reproduction in Camel. J Vet World.

[5] Ali A, Al-sobayil FA, Tharwat M, Al-Hawas A, Ahmed AF (2010). Causes of infertility in female camels (*Camelus dromedarius*) in middle of Saudi Arabia. J Agri Sci and Vet med.

[6] Mohammed HB, Bernard F, Rachid K (2014). Reproductive abnormalities in female camel (*camelus dromedarius*) in Algeria: relationship with age, season, breed and body condition score. J Camel Prac Res.

[7] Abalti A, Bekana M, Woldemeskel M, Lobago F (2006). Female genital tract abnormalities of zebu cattle slaughtered at Bahir-Dar town, North-Western Ethiopia. J Trop Ani Health Prod.

[8] Simenew K, Bekana M, Fikre L, Tilahun Z, Wondu M (2011). Major gross reproductive tract abnormalities in female cattle slaughtered at sululta slaughterhouse in Ethiopia. Glo Vet J.

[9] Mekibib B, Desta T, Tesfaye D. Gross pathological changes in the reproductive tracts of cows slaughtered at two abattoirs in Southern Ethiopia. J Vet Med and Ani Health. 2013;5:46-50.

[10] Shawky AM, Ahmed AT, Mona FI (2004). An abattoir survey of female genital disorders of camels (*Camelus Dromedaries*) in Kalyoubia, Egypt. 1st Annual Conference Moshtohor.

[11] Simenew K, Moa M, Ashenafi F, Tilaye D, Fekadu R (2015). Pathological and bacteriological study on abnormalities of female internal reproductive organ of *Camelus dromedarius* slaughtered at Akaki abattoir, Ethiopia. Am-Eur J Sci Res.

[12] Thrusfield M (1995). Abattoir as source of data veterinary epidemiology 2nd edn.

[13] Buregelt CD (1997). Color atlas of reproductive pathology of domestic animals.

[14] Gebrekidan B, Yilma T, Solmon F (2009). Major causes of slaughter of female cattle in Addis Ababa abattoir enterprise, Ethiopia. Ind J Ani Res.

[15] Chaudhari SU, Pau-Bakko B (2000). Reproductive status, pregnancy wastage and incidence of gross genital abnormalities in cows slaughtered at Maidughuri abattoir, Niger. Pak Vet J.

[16] Tafti A, Darahshiri M (2000). Studies on the uterine abnormalities of slaughtered non-pregnant adult cows. Ind Vet J.

[17] Ali R, Raza AM, Tabbar A, Rasool HM (2006). Pathological studies on reproductive organs of zebu cow. J Agri and Soc Sci.

[18] Ali A, Mehana EE, Ahmed AF, El-Tookhy O, Al-Sobayil A (2011). Ovarian hydrobursitis in female camels (*Camelus dromedarius*): clinical findings, histopathology and fertility after unilateral surgical ablation. Therio..

[19] Al-Afaleq AI, Hegazy AA, Hussein MF, Al-Dughaym AM (2012). Pathological disorders of the female reproductive system in slaughtered camels (*Camelus dromedarius*) in Saudi Arabia. J Comp Clin Path.

[20] Mahmoud MH, Fahad AA, Mostafa MH (2011). Pathologic studies on ovarian abnormalities in Nagas (*Camelus Dromedarius*) in Al-Ahsa, Saudi Arabia. Sci J King Faisal Univ.

[21] Wajid SJ (2015). A pathological abattoir survey of the reproductive tracts of non-pregnant camels (*Camelus dromedaries*) in Iraq. J Phar and Bio Sci.

[22] Mustafa MY, Chaudhry HR, Chaudhry M, Rashid HM, Khan SA (2016). Biometry and pathology of internal genital organs of female Camel (Camelus Dromedarius) in Lahore, Pakistan. Imper J Interdisc Res.

[23] Tibary A, Anouassi A (2001). Retrospective study on an unusual form of ovario-bursal pathology in the camel (*Camelus dromedarius*). Therio..

[24] Gani MO, Amin MM, Alam MG, Kayesh ME, Karim MR (2008). Bacterial flora associated with repeats breeding and uterine infections in dairy cows. Bangl J Vet Med.

[25] Hasan MA, Mamun AA, Uddin AA, Zakir MD, Hassan MZ (2015). Investigation into gyneco-pathological disorders and identification of associated bacteria from the genital organs of cows in Dinajpur, Bangladish. J Adv Vet and Ani Res.

[26] Azawi OI (2008). Postpartum uterine infection in cattle. Ani Repro Sci.

[27] Mshelia GD, Abba Y, Voltaire YA, Akpojie G, Mohammed H (2012). Comparative uterine bacteriology and pathology of camels (*Camelus dromedarius*) and cows in North-Eastern Nigeria. J Comp Clin Path..

[28] Hatipoglu F, Kiran MM, Ortatatli M, Erer H, Çiftçl KM (2002). An abattoir study of genital pathology in cows: Department of Pathology Faculty of veterinary Medicine University of Selcuk. Rev de Méd Vét.

[29] Dalling T, Robertson A, Boddie G, Spruell J (1998). Diseases of camels. The international encyclopedia of veterinary medicine. W Green and Son Edinburgh.

[30] Abbas B, Agab H (2002). A review of camel brucellosis. J Prev Vet Med.

[31] Gwida M, El-Gohary A, Melzer F, Khan I, Rosler U. Brucellosis in camels. J Res Vet Sci. 2011. 10.1016/05002. 10.1016/j.rvsc.2011.05.002 21632084

[32] Tibary A, Fite C (2006). Anouassi, Sghiri a. infectious causes of reproductive loss in camelids. Therio..

[33] Waheed MM, Hamouda MA, Al-Dughaym AM (2009). Uterine histopathological findings of infertile female camels (*Camelus dromedarius*). J Camel Pract Res..

[34] Sendag S, Cetina Y, Alana M, Ilhana F, Eskia F (2008). Cervical leiomyoma in dairy cattle. J Ani Repro Sci.

[35] Timurkaan N, Aydin M, Yilmaz F, Cevik A (2009). Vaginal fibro-leiomyoma in a cow: a case report. J Vet Med.

[36] Arvind S, Adarsh K, Sheikh I, Pankaj S, Rajesh K (2012). Ultrasonographic, surgical, and Histopathological findings of a uterine leiomyoma in a cow. Case Reports in Vet Med.

[37] Kennedy PC, Miller RB. The Female Genital System. In: Pathology of Domestic Animals, Jubb, Kennedy, Palmer Eds., Academic Press, NewYork, vol. 3. 1993. p. 349–470.

[38] Jubb KVF, Kennedy PC, Palmer N (1993). Pathology of domestic animals. In: 4th San Diego, Eds.

[39] Opsomer G, Grohn YT, Hertl J, Coryn M, Deluyker H et al. Risk factors for post-partum ovarian dysfunction in high producing dairy cows in Belgium: A field study. Therio. 2000;53:841-57. 10.1016/S0093-691X(00)00234-X 10730974

[40] Butler W (2003). Energy balance relationships with follicular development, ovulation and fertility in postpartum dairy cows. Livest Prod Sci.

[41] Tibary A, Anouassi A. Ultrasonographic changes of the reproductive tract in the female camel *(Camelus Dromedarius*) during the follicular cycle and pregnancy. J Camel Pract Res. 1996;3:71–90.

[42] Herenda D. Abattoir survey of reproductive organ abnormalities in beef heifers. Canada Vet J. 1987;2:33–6. PMC1680374 17422881

[43] Hegazy AA, Ali A, Al-Aknah M, Ismail S. Studies on pituitary- ovarian axis in the female camel with special reference to cystic and inactive ovaries. J Cam Sci. 2004;1:16–24.

[44] Vanholder T, Opsomer G, Kruif A. Aetiology and pathogenesis of cystic ovarian follicles in dairy cattle: a review. Repr and Nutr Dev. 2006;46:105–19. 10.1051/rnd:2006003 16597418

[45] Roberts SJ. Veterinary Obstetrics and Genital Diseases. 3rd ed. vol. 16. New York: Roberts SJ -Woodstock; 1986. p. 381–59.

[46] Fathalla M, Hailat N, Lafi SQ. An abattoir survey of gross reproductive abnormalities in the bovine genital tract in northern Jordan. Israel J Vet Med. 2000:1–7.

[47] Tibary A, Anouassi A. Reproductive disorders of the female camelidae. In: Tibary A (ed) Theriogenology in *camelidae*: anatomy, physiology, BSE, pathology and artificial breeding: actes editions. Reports of Agronomy and Veterinary Institute Hassan. 1997:317–68.

[48] Alam MGS. Abattoir studies of genital diseases in cows. J Vet Rec. 1984:195–6. 10.1136/vr.114.8.195 6710836

[49] Peter AT, Levine H, Drost M, Bergfelt DR. Compilation of classical and contemporary terminology used to describe morphological aspects of ovarian dynamics in cattle. Therio. 2009:1343–57. 10.1016/j.theriogenology.2008.12.026 19339040

[50] Monaco D, Padalino B, Lacalandra GM. Distinctive features of female reproductive physiology and artificial insemination in the dromedary camel species. Emi J Food and Agri. 2015;27:328–37.

[51] Fetaih H (1991). Some pathological studies on the affections of genital system of she-camel. PhD Thesis, Faculty of veterinary medicine, Suez Canal University.

[52] Tibary A, Anouassi A, Skidmore JA, Adams GP, Recent advances in camelid reproduction (2000). Reproductive disorders in the female camelid. International Veterinary Information Service.

[53] El-Yuguda AD, Abubakar MB, Baba SS, Ngangnou A. Competitive ELISA rinderpest virus antibody in slaughtered camels (Camelus dromedarius): implication for rinderpest virus elimination from Nigeria. Afr J Biomed Res. 2010:83–5.

[54] Tadesse Y, Urge M, Abegaz S, Kurtu YM, Kebede K (2013). Camel and cattle population dynamics and livelihood diversification as a response to climate change in pastoral areas of Ethiopia. Livestock research for rural development.

[55] Tefera M, Abebe G (2012). Camel in Ethiopia. Ethiopian Veterinary Association.

[56] Tibary A (2004). Infertility in female Camelids. Reproductive patterns and examination technique.

[57] Foldi J, Kulcsar M, Pecsi A, Huyghe B, DeSa C (2006). Bacterial complications of postpartum uterine involution in cattle. Ani Repro Sci..

[58] Puck A, Soliame R (2004). Dairy Cattle Husbandry. 2nd edit., Agromisa foundation, Wagenineger, Netherland.

[59] Nicholson MJ, Butterwort MH (1998). A guide to condition scoring of zebu cattle.

[60] Feyissa T, Bekana M (2000). A gross morphological abattoir study of genital organs from female crosses breed and zebu cattle. Israel J Vet Med.

[61] Jenberie S, Awol N, Ayelet G, Gelaye E, Negussie H (2012). Gross and histopathological studies on pulmonary lesions of camel (Camelus dromedarius) slaughtered at Addis Ababa abattoir, Ethiopia. J Trop Ani Health Prod.

[62] Talukder S (2007). Histopathology techniques, tissue processing and staining.

[63] Quinn PJ, Markey BK, Carter ME, Donnelley WJ, Leonard FC (2004). Clinical veterinary microbiology disease.

