# Supplementary material for: Comparative study on lesions of reproductive disorders of cows and female dromedary camels slaughtered at Addis Ababa, Adama and Akaki abattoirs with bacterial isolation and characterization
Source: BMC Vet Res. 2021 Mar 29;17:134. doi: 10.1186/s12917-021-02822-z (PMC8008565; doi:10.1186/s12917-021-02822-z)
Supplement: Supplementary file 1 — Additional file 1: Major reproductive lesions and their macroscopic and microscopic characterization. [file 12917_2021_2822_MOESM1_ESM.docx]

Major reproductive lesions and their macroscopic and microscopic characterization

| Lesion type | Macroscopic characteristics | Microscopic characteristics |
| --- | --- | --- |
| Follicular cyst | Characterized by thin walls, occupy the periphery, the centrum or sometimes almost the entire ovary. They are single or multiple in one or both ovaries. Their diameters ranged from 0.5 -4.5 cm, > 3 cm camel and cows respectively [10,49]. In our case we only considered those follicles which were >3cm. | Zona granulosa disappears entirely or shrink, the theca follicular internal becomes fibrous and the graafian follicles lose their original form [10, 64]. |
| Luteinized cyst | They are smooth, thick-walled, grayish-yellow and smaller in size than follicular cysts with a spherical cavity [49, 57]   - Unlike most of the persistent corpus luteum, luteal cysts lack ovulation papilla (crown). In addition persistent corpus luteum has mushy core area due to the presence of fluid from the degenerating blood clot. | The granulosa cells suffered from vacuolization and  luteinization forming granulosa lutein cells which appeared polyhedral with large vesicular nuclei and vacuolar cytoplasm [10,18] |
| Paraovarian cyst | They appear as fluid-filled anechoic structures and are usually round or oval in shape, occur as a single cystic structure ranged from 1-5 cm in diameter [52] | These cysts consisted of muscular wall contained congested blood vessels and capillaries and lined by simple cuboidal or flattened epithelium. Moreover, pressure atrophy of adjacent ovarian tissues also observed [10,18] |
| Ovarian hydrobursitis | Is a peculiar affection of the ovarian bursa characterized by adhesion, fluid accumulation and encapsulation of the ovary [18,23] | Characterized by degeneration and vacuolation of the mucosal epithelial cells, inflammatory cells infiltration and contained diffuse edema with formation of cyst-like cavities filled with homogenous fluid [6] |
| Inactive ovary | The ovaries are smaller in size, firmer in consistency and contained very small follicles on the surface of non-affected part [10] | Follicles are few in number, not organized; blood vessels are congested in the center of the follicle and also it characterized by excessive proliferation of stroma cells and fibroid connective tissue [11]. |
| Oophoritis | Characterized by discoloration, loss of follicular activity and presence of adhesions [57] | The ovarian medulla was congested and infiltrated by neutrophils, macrophages and lymphocytes, and the ovarian cortex revealed antral and atretic follicles [18]. |
| Pyosalpinx | Characterized by the presence of pus in the fallopian tube. It is a consequence of infection [65] | Characterized by moderate to marked thickening of mucosal layer due to infiltration of neutrophils, macrophages, histocytes and fibrous tissue formation [65] |
| Hemosalpinx | Characterized by thickening the wall of uterine tube due to filling with bloody discharge [26] | Stromal hemorrhage in soft tissue of uterine tube [26] |
| Acute endometritis | Usually detected by the presence of an enlarged uterus with evidence of inflammation such as edema, red-brown watery foul-smelling uterine discharge [38] | Characterized by of infiltration with polymorph nuclear leukocyte (PMNs) in sub-epithelial zone of stratum compactum [66] |
| Chronic endometritis | Characterized by enlarged uterus with congestion of blood vessels [38] | It is characterized by predominantly lymphocytic infiltration some time with presence of plasma cell, macrophage eosinophils or mast cells [26] |
| Cervicitis | Enlarged, edematous and congested mucosa with yellowish or whitish viscous exudates [9] | Congestion of blood vessels, desquamation of the lining epithelium and inflammatory cells infiltration [10] |
| Vaginitis | Vaginal mucosa severely swollen and congested[10] | Congestion of blood vessels, desquamation of the lining epithelium and edema of mucosa with inflammatory cells infiltration[10] |

**References**

64. Lacey M, Rosenberg F. Cystic ovaries in dairy cattle California polytechnic State University. San Luis Obispo. 2010:1–6.

65. Tsianos GP, Stefania MM, George K, Theodor S. Pyosalpinx a Case Report. J Vet Med. 2011;5:546.

66. Powers BE, Johnson LW, Linton LB, Garry F, Smith J. Endometrial biopsy technique and uterine pathologic findings in llamas. J Am Vet Med Assoc. 1990;197:1157–62.
